# Supplementary material for: Do chronic illnesses and poverty go hand in hand?
Source: PLoS One. 2020 Oct 23;15(10):e0241232. doi: 10.1371/journal.pone.0241232 (PMC7584216; doi:10.1371/journal.pone.0241232)
Supplement: S1 Appendix — (DOCX) [file pone.0241232.s001.docx]

**S1 Appendix. Characteristics of Sri Lankan households 2016.**

| **Variable** | **Analytical sample (N=21,756)** | |
| --- | --- | --- |
|  | **% (Means if numerical)** | **Std. deviations** |
| **Socio-economic and demographic characteristics** | |  |
| Chro_ill_patients^b^ | 41.78% |  |
| Males_HH^a^ | 0.4602 | 0.2211 |
| Elders_HH^a^ | 0.1190 | 0.2433 |
| Pr_hh_working^a^ | 0.5839 | 0.3500 |
| Male_headed^b^ | 74.14% |  |
| Head_age^a^ | 52.6272 | 14.0539 |
| Maritalstatus_head^b^ | 77.63% |  |
| Edu_level^a^ | 10.4115 |  |
| **Ethnicity** |  |  |
| Sinhala^b^ | 72.52% |  |
| Sri Lankan Tamil^b^ | 15.07% |  |
| Indian Tamil^b^ | 3.62% |  |
| Sri Lankan Moors^b^ | 8.38% |  |
| Malay^b^ | 0.22% |  |
| Burgher^b^ | 0.13% |  |
| **Religion** |  |  |
| Buddhist^b^ | 68.64% |  |
| Hindu^b^ | 15.30% |  |
| Islam^b^ | 8.59% |  |
| Roman Catholic/Other Christian^b^ | 7.46% |  |
| Health_exp^a^ | 0.0265 | 0.0557 |
| Head_chronic | 26.03% |  |
| **Geographical location** |  |  |
| Sector |  |  |
| Urban^b^ | 15.76% |  |
| Rural^b^ | 79.95% |  |
| District |  |  |
| Colombo^b^ | 9.13% |  |
| Gampaha^b^ | 8.34% |  |
| Kalutara^b^ | 5.26% |  |
| Kandy^b^ | 6.04% |  |
| Matale^b^ | 2.91% |  |
| Nuwara_Eliya^b^ | 3.74% |  |
| Galle^b^ | 5.76% |  |
| Matara^b^ | 5.24% |  |
| Hambantota^b^ | 3.67% |  |
| Jaffna^b^ | 3.16% |  |
| Mannar^b^ | 1.50% |  |
| Vavunia^b^ | 1.58% |  |
| Kilinochchi^b^ | 1.60% |  |
| Batticaloa^b^ | 3.23% |  |
| Ampara^b^ | 3.52% |  |
| Trincomalee^b^ | 2.24% |  |
| Kurunegala^b^ | 7.11% |  |
| Puttalam^b^ | 3.35% |  |
| Anuradhapura^b^ | 3.64% |  |
| Polonnaruwa^b^ | 2.62% |  |
| Badulla^b^ | 3.50% |  |
| Moneragala^b^ | 2.54% |  |
| Ratnapura | 4.79% |  |
| Kegalle | 4.14% |  |
| **Type of chronic illness** |  |  |
| High_sev_diseases^b^ | 34.11% |  |
| Brain_diseases^b^ | 3.25% |  |
| Ent_diseases^b^ | 3.22% |  |
| Otherdiseases^b^ | 4.95% |  |

Source: Author’s calculation based on the DCS [64].

Notes: ^a^ Based on all households that reported every explanatory variable.

^b^ Binary variable.
