# Supplementary material for: Do chronic illnesses and poverty go hand in hand?
Source: PLoS One. 2020 Oct 23;15(10):e0241232. doi: 10.1371/journal.pone.0241232 (PMC7584216; doi:10.1371/journal.pone.0241232)
Supplement: S2 Appendix — (DOCX) [file pone.0241232.s002.docx]

**S2 Appendix. Initial Probit model estimation results for household dataset, Sri Lanka.**

| **Variable** | **Estimate** | **Robust SE** |
| --- | --- | --- |
| Constant | -5.6221 |  |
| **Socio-economic and demographic characteristics** | |  |
| Chro_ill_patients | -0.2532^**^ | 0.1049 |
| Males_HH | -0.2356^***^ | 0.0866 |
| Elders_HH | 0.4851^***^ | 0.1274 |
| Pr_hh_working | -0.2676^***^ | 0.0492 |
| Male_headed | 0.0951 | 0.0536 |
| Head_age | -0.0083^***^ | 0.0017 |
| Maritalstatus_head | 0.1232^**^ | 0.0559 |
| Edu_level | -0.0870^***^ | 0.0046 |
| **Ethnicity** |  |  |
| Sinhala | 3.1605^***^ | 0.1796 |
| Sri Lankan Tamil | 3.4932^***^ | 0.2347 |
| Indian Tamil | 3.3910^***^ | 0.2556 |
| Sri Lankan Moors | 3.3588^***^ | 0.2728 |
| Malay | 3.7928^***^ | 0.4361 |
| Burgher | 3.3532^***^ | 0.2756 |
| **Religion** |  |  |
| Buddhist | 2.5389^***^ | 0.2314 |
| Hindu | 2.4353^***^ | 0.2315 |
| Islam | 2.3559^***^ | 0.2534 |
| Roman Catholic/Other Christian | 2.3598^***^ | . |
| Health_exp | -3.4044^**^ | 0.6820 |
| Head_chronic | -0.1252^*^ | 0.0588 |
| **Geographical location** | |  |
| Urban | -0.1480 | 0.1332 |
| Rural | 0.1045^**^ | 0.1146 |
| Colombo | -1.0472^***^ | 0.1638 |
| Gampaha | -0.8764^***^ | 0.1456 |
| Kalutara | -0.7371^***^ | 0.1424 |
| Kandy | -0.4430^***^ | 0.1304 |
| Matale | -0.5710^***^ | 0.1489 |
| Nuwara_Eliya | -0.5819^***^ | 0.1522 |
| Galle | -0.6020^***^ | 0.1400 |
| Matara | -0.2766^**^ | 0.1325 |
| Hambantota | -0.9407^***^ | 0.1641 |
| Jaffna | -0.3948^***^ | 0.1189 |
| Mannar | -1.3704^***^ | 0.2485 |
| Vavunia | -1.3130^***^ | 0.2204 |
| Kilinochchi | -0.0393 | 0.1236 |
| Batticaloa | -0.4580^***^ | 0.1168 |
| Ampara | -0.8538^***^ | 0.1444 |
| Trincomalee | -0.3866^***^ | 0.1309 |
| Kurunegala | -0.6738^***^ | 0.1361 |
| Puttalam | -0.7626^***^ | 0.1578 |
| Anuradhapura | -0.5312^***^ | 0.1459 |
| Polonnaruwa | -0.8435^***^ | 0.1719 |
| Badulla | -0.1606 | 0.1377 |
| Moneragala | -0.3473^**^ | 0.1439 |
| Ratnapura | -0.3141^**^ | 0.1324 |
| Kegalle | -0.2925^**^ | 0.1350 |
| **Type of chronic illness** | |  |
| High_sev_diseases | 0.2207^**^ | 0.0997 |
| Brain_diseases | 0.5333^***^ | 0.0959 |
| Ent_diseases | 0.2287^*^ | 0.1177 |
| Otherdiseases | 0.2586^**^ | 0.1038 |
| Area under ROC curve | 0.7969 | |
| Pseudo R^2^ | 0.2439 | |
| Log likelihood | -3229.9708 | |
| No. of observation | 20,696 | |

Source: Author’s calculation based on the DCS [64].

Note: ***significant at the 1% level; ** significant at the 5% level; significant at the 10% level.

Variable ‘Burgher’ dropped with 26 observation because of estimability.
